# Investigating the Activity Spectrum for Ring-Substituted 8-Hydroxyquinolines

**DOI:** 10.3390/molecules15010288

**Published:** 2010-01-12

**Authors:** Robert Musiol, Josef Jampilek, Jacek E. Nycz, Matus Pesko, James Carroll, Katarina Kralova, Marcela Vejsova, Jim O'Mahony, Aidan Coffey, Anna Mrozek, Jaroslaw Polanski

**Affiliations:** 1 Institute of Chemistry, University of Silesia, Szkolna 9, 40007 Katowice, Poland; 2 Zentiva k.s., U kabelovny 130, 102 37 Prague, Czech Republic; 3 Department of Chemical Drugs, Faculty of Pharmacy, University of Veterinary and Pharmaceutical Sciences, Palackeho 1/3, 61242 Brno, Czech Republic; 4 Department of Ecosozology and Physiotactics, Faculty of Natural Sciences, Comenius University, Mlynska dolina Ch-2, 84215 Bratislava, Slovakia; 5 Department of Biological Sciences, Cork Institute of Technology, Bishopstown, Cork, Ireland; 6 Institute of Chemistry, Faculty of Natural Sciences, Comenius University, Mlynska dolina Ch-2, 84215 Bratislava, Slovakia; 7 Department of Biological and Medical Sciences, Faculty of Pharmacy in Hradec Kralove, Charles University in Prague, Heyrovskeho 1203, 500 05 Hradec Kralove, Czech Republic

**Keywords:** quinolines, lipophilicity, PET inhibition, spinach chloroplasts, *in vitro* antifungal activity, *in vitro* antimycobacterial activity

## Abstract

In this study, a series of fourteen ring-substituted 8-hydroxyquinoline derivatives were prepared. The synthesis procedures are presented. The compounds were analyzed using RP-HPLC to determine lipophilicity. They were tested for their activity related to inhibition of photosynthetic electron transport (PET) in spinach (*Spinacia oleracea* L.) chloroplasts. Primary *in vitro* screening of the synthesized compounds was also performed against four mycobacterial strains and against eight fungal strains. Several compounds showed biological activity comparable with or higher than the standards isoniazid or fluconazole. For all the compounds, the relationships between the lipophilicity and the chemical structure of the studied compounds are discussed.

## 1. Introduction

A quinoline moiety is present in many classes of biologically-active compounds. A number of them have been used clinically as antifungal, antibacterial and antiprotozoic drugs [1,2], as well as antituberculotic agents [3,4,5]. Some quinoline-based compounds also show antineoplastic, antiasthmatic and antiplatelet activity [6,7,8,9,10,11]. A series of compounds derived from 8-hydroxyquinoline and styrylquinoline derivatives were recently synthesized as potential HIV-1 integrase inhibitors [12,13,14,15]. These compounds showed a significant similarity to some novel antifungal agents, namely homoallylamines. [16]. Our previous study dealing with 8-hydroxyquinoline and styrylquinoline derivatives showed that they could also possess strong antifungal activity [17,18]. According to the results reported recently, some new hydroxyquinoline derivatives also possess interesting herbicidal activities [17,19,20,21,22]. In addition, some of the investigated quinoline derivatives also showed antineoplastic activity [19,23].

Tuberculosis is a worldwide pandemic. About 1/3 of the world's population is infected with *Mycobacterium tuberculosis*, and every year almost 2 million people die as a result [24]. The *Mycobacterium* genus is composed of the *M. tuberculosis* complex and other species known as nontuberculous mycobacteria (NTM). In recent decades, the decrease in the prevalence of tuberculosis in developed countries has resulted in the increase in the proportion of diseases caused by NTM [25]. Among these species, the *M. avium* complex (MAC) has emerged as a major human pathogen, being a common cause of disseminated disease and death in patients with HIV/AIDS [26].

Chronic pulmonary disease is the most common clinical manifestation among the diseases caused by NTM, and the most common pathogens are the species belonging to the MAC, followed by *M. kansasii*. The clinical characteristics of NTM-related pulmonary disease are, in many cases, extremely similar to those of tuberculosis. Other clinical manifestations are caused principally by *M. fortuitum, M. smegmatis* and *M. abscessus* due to peritoneal infection as a result of catheterization, postsurgical infections, such as those following mammoplasty and heart transplant, as well as those following invasive procedures [27]. The above mentioned non-tuberculous strains are sometimes resistant to commonly used drugs (isoniazid, rifampicin, pyrazinamide) and other anti-tuberculous drugs [24], therefore systematic development of new effective compounds is necessary. Similarly, the discovery of new drugs for the treatment of systemic mycoses with novel modes of action due to the rapid growth of the immunocompromised patient population and development of resistance to the present azole therapies, and high toxicity of polyenes [28] is indispensable. It should be stressed that hydroxyquinoline and its derivatives were introduced as antifungal or antimycobacterial agents in clinical practice and novel compounds of this type are still investigated [3,4,5,29,30].

Over 50% of commercially available herbicides act by reversibly binding to photosystem II (PS II), a membrane-protein complex in the thylakoid membranes which catalyses the oxidation of water and the reduction of plastoquinone [31] and thereby inhibit photosynthesis [32,33,34]. Some organic compounds, e.g., substituted benzanilides [35] or substituted anilides of 2,6-disubstituted pyridine-4-thiocarboxamides [36] or pyrazine-2-carboxylic acids [37] were found to interact with tyrosine radicals Tyr_Z_ and Tyr_D_ which are situated in D_1_ and D_2_ proteins on the donor side of PS II and due to this interaction the photosynthetic electron transport is interrupted.

This is a follow-up paper to our previous articles [12,13,14,15,17,18,19,20,21,22,23] dealing with syntheses and biological activities of ring-substituted quinoline derivatives. On the basis of formerly described azanaphtalenes we tried to search for new modifications of quinoline moiety that can trigger interesting biological activity.

Primary *in vitro* screening of the synthesized compounds was performed against four mycobacterial strains and against eight fungal strains. The compounds were also tested for their photosynthesis-inhibiting activity (the inhibition of photosynthetic electron transport) in spinach chloroplasts (*Spinacia oleracea* L.). Relationships among the structure and *in vitro* antimicrobial activities or/and inhibitory activity related to inhibition of photosynthetic electron transport (PET) in spinach chloroplasts of the new compounds are discussed.

## 2. Results and Discussion

### 2.1. Chemistry

**Scheme 1:**
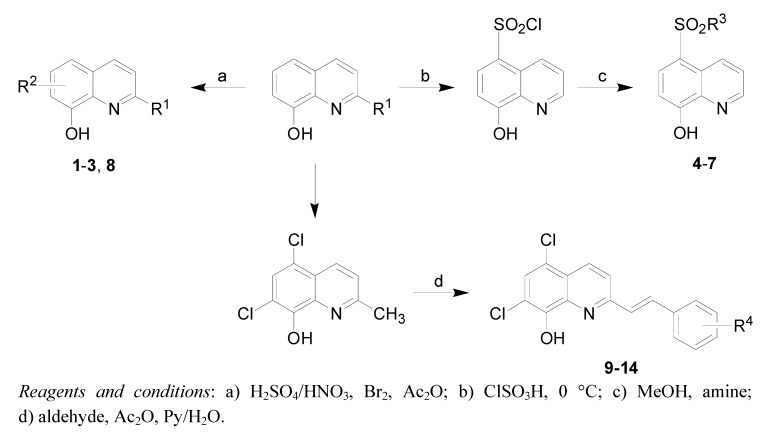
Synthesis of studied compounds.

All studied compounds were prepared according to Scheme 1. Compounds **1**-**3** were obtained according to a previously described procedure [14]. Sulfonamides **4**-**7** were obtained from 8-hydroxyquinoline through chlorosulfonation and amination. Compound **8** was obtained from commercially available 8-hydroxy-2-aminoquinoline by acylation in acetic anhydride. Styryquinolines **9**-**14** were obtained from 5,7-dichloro-8-hydroxyquinaldine and the appropriate aldehyde in a two-step reaction in acetic anhydride followed by pyridine/water.

### 2.2. Lipophilicity

Many low molecular weight drugs cross biological membranes through passive transport, which strongly depends on their lipophilicity. Lipophilicity is a property that has a major effect on absorption, distribution, metabolism, excretion, and toxicity (ADME/Tox) properties as well as pharmacological activity. Lipophilicity has been studied and applied as an important drug property for decades [38].

**Table 1:** Comparison of the calculated lipophilicities (log *P*/Clog *P*) with the determined log *k* values.

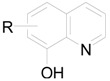				
Comp.	R	log *k*	log *P*/Clog *P*	log *P*
			ChemOffice	ACD/LogP
**1**	5-NO_2_	0.5695	1.69 / 2.0836	2.00 ± 0.32
**2**	5-SO_3_H-7-NO_2_	0.1479	0.37 / -0.703	1.70 ± 0.88
**3**	5-SO_3_H-7-Br	0.3786	1.72 / -0.004	2.39 ± 0.91
**4**	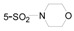	0.3293	0.61 / 1.43775	1.19 ± 0.81
**5**	5-SO_2_NHCH(CH_3_)_2_	0.3373	2.17 / 2.5697	1.97 ± 0.78
**6**	5-SO_2_NHC_2_H_4_Ph	0.3349	3.17 / 3.83597	3.29 ± 0.79
**7**	5-SO_2_NHC_4_H_8_Ph	0.3902	4.09 / 4.74397	4.18 ± 0.78
**8**	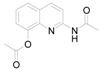	0.3583	1.33 / 0.93375	0.52 ± 0.73
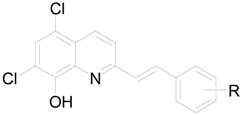				
**Comp.**	**R**	**log *k***	**log *P*/Clog *P***	**log *P***
			ChemOffice	ACD/LogP
**9**	2-OH	1.5854	5.07 / 5.36425	5.62 ± 0.35
**10**	4-OH	1.5866	5.07 / 5.36425	6.37 ± 0.36
**11**	2,4-OH	1.5858	4.68 / 4.69725	5.65 ± 0.37
**12**	3,5-OH	1.5867	4.68 / 4.69725	6.20 ± 0.37
**13**	2-OH-5-OAc	1.5864	4.66 / 4.89345	4.87 ± 0.36
**14**	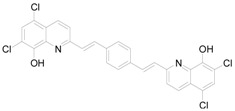	2.5760	8.88 / 9.92051	10.50 ± 0.39

**Figure 1:**
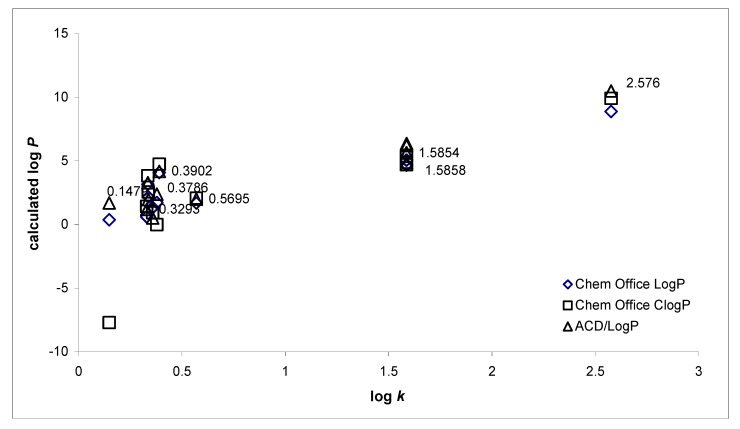
Comparison of the computed log *P*/Clog *P* values using two programs with the calculated log *k* values.

Hydrophobicities (log *P*/Clog *P*) of the compounds **1**-**14** were calculated using two commercially available programs (ChemDraw Ultra 10.0 and ACD/LogP) and also measured by means of the RP-HPLC determination of capacity factors *k* with subsequent calculation of log *k*. The procedure was performed under isocratic conditions with methanol as an organic modifier in the mobile phase using an end-capped non-polar C_18_ stationary RP column. The ChemDraw program did not resolve various lipophilicity values of individual positional isomers, that is, the same log *P*/Clog *P* data were calculated for **9**/**10** and **11**/**12**. The results are shown in Table 1 and illustrated in Figure 1.

The results obtained with all the compounds show that the experimentally-determined lipophilicities (log *k*) of compounds **1**-**14** are lower than those indicated by the calculated log *P*/Clog *P*, as shown in Figure 1. The results indicate that experimentally-determined log *k* values correlate relatively poorly with the calculated values. The correlation factors R^2^ yielded 0.83 for ChemOffice log *P*; 0.86 for ACD/LogP and 0.55 for ChemOffice Clog *P* respectively.

These facts are probably caused by intramolecular interactions. As expected, compound **14** showed the highest lipophilicity, while compound **2** exhibited the lowest. The presence of the sulfonic acid moiety decreased the lipophilicity. Compound **6** showed less hydrophobicity compared with **5**, contrary to the lipophilicity results calculated by software. This observation has been made previously [19]. Interesting results were obtained with subseries **9**-**13**. Comparing the lipophilicity data log *k* of both OH-monosubstituted isomers **9** and **10**, it can be stated that 4-hydroxy derivative **10** possessed higher hydrophobicity than 2-hydroxy isomer **9**. This fact was predicted only by ACD/LogP. When OH-disubstituted compounds were compared, it was found that strong intramolecular interactions play a significant role. Hydrophobicity increased in the following order: **11** (2,4-OH) < **12** (3,5-OH). These results were also predicted by ACD/LogP.

It can be assumed, that log *k* values specify lipophilicity within the individual series of studied compounds. Calculated log *P* data (ChemDraw) of compounds **1**-**8** corresponded with experimentally-determined log *k*, while for compounds **9**-**14** there was better agreement between the calculated log *P* data (ACD/LogP) and experimentally-determined log *k*.

### 2.3. Biological activities

The compounds under investigation could be divided into two groups based on their chemical structure. Group 1 included compounds **1**-**8**, and Group 2 compounds **9**-**14**. The compounds showed a wide range of biological activities and structure-activity relationships observations are interesting. All the results are shown in Table 2 and Table 3.

**Table 2:** IC_80_ values related to PET inhibition in spinach chloroplasts in comparison with 3-(3,4-dichlorophenyl)-1,1-dimethylurea (DCMU) standard and *in vitro* antifungal activity (IC_80_) of compounds **1**-**10**, **12** and **14** compared with fluconazole (FLU) standard. (ND = not determined due to insufficient solubility of the compound).

**Comp.**	**PET reductionIC_50_ [** **μmol/L]**	**MIC [µmol/L]**							
		CA*^a^*	CT*^a^*	CK*^a^*	CG*^a^*	TB*^a^*	AF*^b^*	AC*^b^*	TM*^b^*
		*24h*	*24h*	*24h*	*24h*	*24h*	*24h*	*24h*	*72h*
		*48h*	*48h*	*48h*	*48h*	*48h*	*48h*	*48h*	*120h*
**1**	78.0	>500	>500	>500	>500	>500	>500	>500	>500
		>500	>500	>500	>500	>500	>500	>500	>500
**2**	ND	>250	>250	>250	>250	>250	>250	>250	>250
		>250	>250	>250	>250	>250	>250	>250	>250
**3**	33.5	0.77	1.95	3.90	1.95	3.90	0.77	7.80	1.95
		1.95	3.90	3.90	3.90	3.90	1.95	7.80	1.95
**4**	ND	125	500	250	500	500	500	125	250
		500	500	500	500	500	500	250	250
**5**	54.4	125	250	250	125	250	250	125	62.50
		250	250	250	250	500	250	125	125
**6**	304	125	125	125	125	125	125	125	125
		125	125	125	125	125	125	125	125
**7**	616	>125	>125	>125	>125	>125	>125	>125	>125
		>125	>125	>125	>125	>125	>125	>125	>125
**8**	306	15.60	31.25	62.50	31.25	62.50	62.50	62.50	62.50
		62.50	31.25	62.50	62.50	125	62.50	62.50	62.50
**9**	ND	>125	>125	>125	>125	>125	125	62.50	62.50
		>125	>125	>125	>125	>125	125	125	62.50
**10**	660	125	125	125	125	125	125	31.25	15.62
		>125	>125	>125	>125	>125	125	62.50	62.50
**12**	341	>125	>125	>125	>125	>125	125	62.50	15.62
		>125	>125	>125	>125	>125	125	62.50	15.62
**14**	334	0.98	1.95	0.98	0.49	3.90	3.90	15.62	3.90
		3.90	7.81	1.95	1.95	15.62	15.62	31.25	7.81
**DCMU**	1.9	–	–	–	–	–	–	–	–
**FLU**	–	0.06	0.12	3.91	0.98	0.24	>125	>125	1.95
		0.12	>125	15.62	3.91	0.48	>125	>125	3.91

The MIC determination was performed according to the CLSI reference protocol: *^a ^*M27-A2 for yeasts (IC_80_ value) and *^b ^*M38-A for moulds (IC_50_ value).

#### 2.3.1. Inhibition of photosynthetic electron transport (PET) in spinach chloroplasts

Quinoline derivatives **1**-**10**, **12** and **14** showed a wide range of activity related to inhibition of photosynthetic electron transport (PET) in spinach chloroplasts, see Table 2. Three compounds showed moderate inhibitory IC_50_ values: 33.5 µmol/L (**3**), 54.4 µmol/L (**5**) and 78.0 µmol/L (**1**) while the activity of all the other studied compounds was very low. PET inhibition by **2**, **4** and **9** could not be determined due to precipitation of the compounds during the experiments.

The highest PET inhibition was shown by compounds **3**, **5** and **1** with suitable aqueous solubility as well as lipophilicity. The sulfonic acid moiety contributed to enhanced aqueous solubility whereas a 7-bromo moiety (compound **3**) increased the lipophilicity. Herbicidal effects of organic compounds with bromo or nitro substituents were observed previously [39]. Bulkiness and rigidity of the chain in the R substituent play a fundamental role in the series of sulfonamides **4**-**7**, in addition to lipophilicity and water-solubility. The least lipophilic morpholine derivative **4** did not show any activity. On the other hand, phenylbutyl substitution in compound **7** is probably too bulky for it to reach the site of action in the photosynthetic electron transport chain and thus the inhibitory activity of this compound is low. Within the sulfonamide series the isopropyl substituent was the most advantageous from the aspect of effective PET inhibition and further lipophilicity increase caused reduction of inhibitory activity. On the other hand, great differences in the activity of compounds characterized with rather small differences in the lipophilicity (the log *k* values of compounds belonging to Group 1) indicate that the position of substituents and their electronic Hammett's parameters σ (especially electron-withdrawing effect) seem to be also important for biological activity. Lipophilicity of the discussed compounds is only a secondary parameter influencing PET-inhibiting activity. In general, Group 2 compounds exhibited only slight inhibition of PET and IC_50_ values determined for three compounds do not enable conclusions about structure-activity relationships.

#### 2.3.2. *In vitro* antifungal susceptibility testing

Quinoline derivatives **1**-**10**, **12** and **14** were tested for their *in vitro* antifungal activity. The results are shown in Table 2. Two compounds **3** and **14** showed very high antifungal activity, comparable or higher than the standard fluconazole.

Generally, Group 1 showed lower biological activity than Group 2, although compound **3** demonstrated high activity against all fungal strains. Compounds **1**, **2**, **4**-**7** did not show any antifungal activity. Compound **8** showed only moderate activity especially against *Candida albicans* ATCC 44859.

Unfortunately due to general low activity, no thorough structure-activity relationships (SAR) can be established. Nevertheless, an interesting relationship can be observed. According to the results presented in Table 1 and Table 2 it can be concluded that bulky substituents and low lipophilicity decreased antifungal activity. A bromo moiety (compound **3**) seems to be very important for high antifungal effect, as was reported recently [17]. Contrary to all expectations [18], compounds with a nitro moiety (*i.e*., **1**, **2**) did not show any activity. It can be concluded that relatively minor differences in lipophilicity were correlated with major differences in the activity of compounds. This suggests that for biological activity, the position of substituents and their electronic Hammett's parameters σ seem to be important. Similar observations were made with the PET-inhibiting activity.

Compound **14** showed the highest antifungal activity within Group 2. The compounds **9**-**12** showed only a moderate activity especially against *Absidia corymbifera* 272 and *Trichophyton mentagrophytes* 445. Contrary to all expectations [18], **9** (2-OH substitution) possessed less activity than **10 **and **12** (4-OH or 3,5-OH respectively). This fact is probably caused by low solubility of **9**. Lipophilicity is probably an important parameter influencing the activity.

#### 2.3.3. *In vitro* antimycobacterial evaluation

Six compounds within Group 2 **9**-**14** were evaluated for their *in vitro* antimycobacterial activity against four mycobacterial strains. The results are shown in Table 3. Compound **12** (3,5-OH) did not exhibit any significant antimycobacterial activity and compounds **9**-**11**, **13** showed only medium and/or moderate activities. Nevertheless, compound **14** had an interesting MIC especially against *M. smegmatis*, *M. kansasii* and *M. absessus*. This compound was more active than the standard pyrazinamide (PZA) and in case of *M smegmatis*, its activity was comparable with the standard isoniazid (INH).

**Table 3:** Antimycobacterial activity MIC/IC_90_ [µg/mL] of compounds **9**-**14** in comparison with the standards, pyrazinamide (PZA) and isoniazid (INH).

**Comp.**	**MIC/IC_90_ [µg/mL]**			
	*M. smegmatis*	*M. absessus*	*M. kansasii*	*M. avium *complex
**9**	100	>300	>300	>300
**10**	150	>300	>300	>300
**11**	100	>100	>100	>100
**12**	>100	>300	>300	>300
**13**	100	>300	>300	>300
**14**	40	130	40	>300
**PZA**	>100	>100	>100	>100
**INH**	39	>100	<10	<10

Due to medium and/or moderate activity of the majority of evaluated compounds, it is difficult todetermine simple structure-activity relationships. According to Table 1 and Table 3, it can be concluded that lipophilicity (log *k*) and other physico-chemical parameters only influence secondary characteristics of the compounds.

## 3. Conclusions

A series of fourteen ring-substituted 8-hydroxyquinolines were prepared and characterized. Compounds **1**,**3**-**14** are original new structures. All the prepared quinoline derivatives were analyzed using a RP-HPLC method for the measurement of lipophilicity and their lipophilicity was determined. The prepared compounds were tested for their antifungal and antimycobacterial activity and for their activity related to the inhibition of photosynthetic electron transport (PET) in spinach chloroplasts (*Spinacia oleracea* L.). 7-Bromo-8-hydroxyquinoline-5-sulfonic acid (**3**) and 5,7-dichloro-2-(2-{4-[2-(5,7-dichloro-8-hydroxy-quinolin-2-yl)vinyl]phenyl}vinyl)quinolin-8-ol (**14**) possessed the highest *in vitro* antifungal activity and **3** and 8-hydroxyquinoline-5-sulfonic acid isobutylamide (**5**) showed the highest PET-inhibiting activity. Compound **14** showed the highest antimycobacterial activity. In general, compounds **3** and **14** showed the highest inhibitory effects within the series.

## 4. Experimental

### 4.1. General

All reagents were purchased from Aldrich. Kieselgel 60, 0.040-0.063 mm (Merck, Darmstadt, Germany) was used for column chromatography. TLC experiments were performed on alumina-backed silica gel 40 F254 plates (Merck, Darmstadt, Germany). The plates were illuminated under UV (254 nm) and evaluated in iodine vapour. The melting points were determined on Boetius PHMK 05 (VEB Kombinat Nagema, Radebeul, Germany) and are uncorrected. Elemental analyses (E.A.) were carried out on an automatic Perkin-Elmer 240 microanalyser (Boston, MA, USA) for C, H, N and are within 0.4% of theoretical values. The purity of the final compounds was checked by the HPLC separation module Waters Alliance 2695 XE (Waters Corp., Milford, MA, USA). The detection wavelength 210 nm was chosen. The peaks in the chromatogram of the solvent (blank) were deducted from the peaks in the chromatogram of the sample solution. The purity of individual compounds was determined from the area peaks in the chromatogram of the sample solution. UV spectra (λ, nm) were determined on a Waters Photodiode Array Detector 2996 (Waters Corp., Milford, MA, USA) in ca 6×10^-4^ mol methanolic solution and log ε (the logarithm of molar absorption coefficient ε) was calculated for the absolute maximum λ_max_ of individual target compounds. All ^1^H-NMR spectra were recorded on a Bruker AM-500 (499.95 MHz for ^1^H), and ^13^C-NMR spectra were recorded on a Bruker AM-400 (100 MHz) instrument (Bruker BioSpin Corp., Germany). Chemicals shifts are reported in ppm (δ) with reference to internal Si(CH_3_)_4_, when diffused easily exchangeable signals are omitted.

### 4.2. Synthesis

*5-Nitroquinolin-8-ol* (**1**). Yield: 25% of a yellow crystalline compound; Mp. 179-180 °C; HPLC purity 98.09%; UV (nm), λ_max_/log ε: 398.4/3.67) [14].

*8-Hydroxy-7-nitroquinoline-5-sulfonic acid* (**2**). Yield: 54% of a bright yellow crystalline compound; Mp. 265 °C; HPLC purity 97.97%; UV (nm), λ_max_/log ε: 427.9/3.71) [40].

*7-Bromo-8-hydroxyquinoline-5-sulfonic acid* (**3**). Yield: 25% of a beige crystalline compound; Mp. 280 °C; HPLC purity 98.32%; UV (nm), λ_max_/log ε: 264.2/3.69) [14].

*5-(Morpholine-4-sulfonyl)quinolin-8-ol* (**4**). 8-Hydroxyquinoline-5-sulfonyl chloride (2.4 g, 0.01 mol) was dissolved in dry MeOH (30 mL) and heated to reflux. Then morpholine (1.75 mL, 0.02 mol) was added dropwise. After 2 h of heating the resulted mixture was concentrated *in vacuo* and the resulting brown oil put into dichloromethane (45 mL). The orange precipitate formed was filtered off and recrystallized from MeOH. Yield 73% of a light yellow crystalline compound; Mp. 172 °C; E.A. Calcd.: C 54.53%, H 5.23%, N 9.08%; found: C 54.26%, H 5.31%, N 9.27%; HPLC purity 97.89%; UV (nm), λ_max_/log ε: 264.0/3.65; ^1^H-NMR (DMSO-*d*_6_), δ: 2.52 (s, 1H), 3.03 (t, *J* = 4.6 Hz, 4H), 3.70 (t, *J* = 4.7 Hz, 4H), 6.95 (d, *J* = 7.9 Hz, 1H), 7.80 (d, *J* = 7.9 Hz, 1H), 9.05 (d, *J* = 8.4 Hz, 1H), 9.10 (d, *J* = 8.4 Hz, 2H).

*8-Hydroxyquinoline-5-sulfonic acid isobutylamide* (**5**). 8-Hydroxyquinoline-5-sulfonyl chloride (2.2 g, 0.01 mol) was dissolved in dry MeOH (30 mL) and heated to reflux. Then 2-butylamine (2.02 mL, 0.02 mol) was added dropwise. After 2 h of heating a yellow solid was precipitated from the reaction mixture. After cooling the solid was filtered and crystallized from MeOH. Yield 50% of a yellow crystalline compound; Mp. 151 °C; E.A. Calcd.: C 55.70%, H 5.75%, N 9.99%; found: C 55.67%, H 5.63%, N 9.84%; HPLC purity 97.29%; UV (nm), λ_max_/log ε: 265.2/3.64; ^1^H-NMR (DMSO-*d*_6_), δ: 0.85 (t, *J* = 7.4 Hz, 3H), 1.05 (d, *J* = 6.4 Hz, 1H), 1.50 (m, 2H), 2.50 (s, 1H), 2.95 (m, 1H), 6.95 (d, *J* = 8.0 Hz, 1H), 7.80 (d, *J* = 7.9 Hz, 1H), 9.05 (d, *J* = 7.6 Hz, 1H), 9.10 (d, *J* = 7.3 Hz, 2H).

*8-Hydroxyquinoline-5-sulfonic acid (2-phenylethyl)amide* (**6**). Obtained in similar way to **4** from 8-hydroxyquinoline-5-sulfonyl chloride and phenylethylamine. Yield: 25% of a yellow crystalline compound; Mp. 104 °C; E.A. Calcd.: C 62.18%, H 4.91%, N 8.53%; found: C 62.32%, H 4.86%, N 8.71%. HPLC purity 97.37%; UV (nm), λ_max_/log ε: 265.5/3.68; ^1^H-NMR (DMSO-*d*_6_), δ: 1.38 (d, *J* = 6.7 Hz, 2H), 2.50 (t, *J* = 1.6 Hz, 1H), 2.66 (s, 1H), 6.90 (d, *J* = 7.8 Hz, 1H), 7.30-7.41 (m, 2H), 7.72 (d, *J* = 8.0 Hz, 2H), 8.96 (d, *J* = 8.8 Hz, 1H), 7.42-7.44 (m, 5H), 7.25 (d, *J* = 7.5 Hz, 1H).

*8-Hydroxyquinoline-5-sulfonic acid (4-phenylbutyl)amide* (**7**). Obtained in similar way to **4** from 8-hydroxyquinoline-5-sulfonyl chloride and 4-phenylbuthylamine. Yield: 55% of a yellow crystalline compound; Mp. 125 °C; E.A. Calcd: C 64.84%, H 5.99%, N 7.56%; found: C 65.02%, H 5.85%, N 7.69%; HPLC purity 98.60%; UV (nm), λ_max_/log ε: 265.3/3.68; ^1^H-NMR (DMSO-*d*_6_), δ: 1.44-1.58 (m, 2H), 2.49 (s, 1H), 2.67 (t, *J* = 6.8 Hz, 1H), 6.95 (d, *J* = 7.9 Hz, 1H), 2.56 (t, *J* = 7.2 Hz, 2H), 7.18 (d, *J* = 6.8 Hz, 1H), 7.24 (t, *J* = 7.0 Hz, 5H), 7.54-7.58 (m, 2H), 7.82 (d, *J* = 7.8 Hz, 1H), 9.06 (d, *J* = 8.5 Hz, 1H), 9.12 (d, *J* = 8.5 Hz, 2H).

*2-Acetylaminoquinolin-8-yl acetate* (**8**). 2-Amino-8-hydroxyquinoline (2.5 g, 0.01 mol) and acetic anhydride (4 g, 3 mL, 0.04 mol) was mixed and heated in the microwave reactor at the boiling point for 5 min, then evaporated until dryness. Crude product was recrystalized from EtOH and dried. Yield: 53% of a white crystalline compound; Mp. 120-126 °C; HPLC purity 99.69%; UV (nm), λ_max_/log ε: 258.3/3.69; E.A. Calcd: C 63.93%, H 4.95%, N 11.47%; found: C 63.82%, H 4.56%, N 11.25%; ^1^H-NMR (DMSO-*d*_6_), δ: 2.19 (s, 3H), 2.22 (s, 3H), 7.45 (m, 2H), 7.61 (d, *J* = 8.65Hz, 1H), 7.8 (t, *J* = 9.5Hz, 1H), 8.37 (d, *J* = 9.0Hz, 1H), 10.5 (s, 1H).

#### 4.2.1. General method for synthesis of compounds **9-14**.

To quinaldine (0.01 mol) in acetic anhydride (30 mL) an appropriate aldehyde (0.04 mol) was added and resulting mixture was heated under reflux for 24 h. Then, the liquid was evaporated *in vacuo*, pyridine (30 mL) and water (10 mL) were added and the mixture further heated for 3 h under reflux. Then mixture was evaporated to dryness and solid was taken up in dichloromethane and filtered. Crude product was recrystalized from EtOH and dried.

*5,7-Dichloro-2-[2-(2-hydroxyphenyl)vinyl]quinolin-8-ol* (**9**). Yield 87% of a beige crystalline compound; Mp. 141 °C; E.A. Calcd.: C 61.47%, H 3.34%, N 4.22%; found: C 61.87%, H 3.05%, N 4.12%; HPLC purity 98.10%; UV (nm), λ_max_/log ε: 309.9/3.61; ^1^H-NMR (DMSO-*d*_6_), δ: 6.88 (t, *J* = 7.4 Hz, 1H, phenyl), 6.95 (d, *J* = 8.1 Hz, 1H, phenyl), 7.20 (t, *J* = 7.7 Hz, 1H, phenyl), 7.57 (d, *J* = 16.3 Hz, 1H, vinyl), 7.61 (d, *J* = 7.7 Hz, 1H, phenyl), 7.68 (s, 1H, quinaldine), 7.93 (d, *J* = 8.8 Hz, 1H, quinaldine), 8.23 (d, *J* = 16.3 Hz, 1H, vinyl), 8.39 (d, *J* = 8.8 Hz, 1H, quinaldine), 10.08 (s, 1H, OH), 10.46 (bs, 1H, OH); ^13^C-NMR (DMSO-*d*_6_), δ: 116.01, 116.66, 119.63, 119.93, 122.19, 123.40, 123.94, 127.22, 127.84, 129.10, 130.56, 133.06, 133.55, 139.35, 149.11, 156.57, 156.74.

*5,7-Dichloro-2-[2-(4-hydroxyphenyl)vinyl]quinolin-8-ol* (**10**). Yield 86% of a beige crystalline compound; Mp. 198 °C; E.A. Calcd.: C 61.47%, H 3.34%, N 4.22%; found: C 61.54%, H 3.32%, N 4.28%; HPLC purity 98.52%; UV (nm), λ_max_/log ε: 309.9/3.58; ^1^H-NMR (DMSO-*d*_6_), δ: 6.85 (d, *J* = 7.2 Hz, 2H, phenyl), 7.28 (d, *J* = 16.0 Hz, 1H, vinyl), 7.56 (d, *J* = 7.3 Hz, 2H, phenyl), 7.69 (d, *J* = 1.7 Hz, 1H, quinaldine), 7.86 (dd, *J* = 1.2 Hz, *J* = 8.8 Hz, 1H, quinaldine), 8.18 (d, *J* = 16.1 Hz, 1H, vinyl), 8.40 (dd, *J* = 1.3 Hz, *J* = 8.8 Hz, 1H, quinaldine), 9.84 (s, 1H, OH), 10.30 (bs, 1H, OH); ^13^C-NMR (DMSO-*d*_6_), δ: 115.17, 115.73, 119.02, 122.07, 123.19, 123.41, 126.41, 127.14, 128.97, 132.97, 136.53, 138.66, 148.36, 155.67, 158.52.

*5,7-Dichloro-2-[2-(2,4-dihydroxyphenyl)vinyl]quinolin-8-ol* (**11**). Yield 83% of a beige crystalline compound; Mp. 102-1030 °C; HPLC purity 98.96%; UV (nm), λ_max_/log ε: 321.9/3.65 [41].

*5,7-Dichloro-2-[2-(3,5-dihydroxyphenyl)vinyl]quinolin-8-ol* (**12**). Yield 93% of a beige crystalline compound; Mp. 206 °C; HPLC purity 98.59%; UV (nm), λ_max_/log ε: 314.5/3.68 [42].

*5,7-Dichloro-2-[2-(2-hydroxy-5-acetoxyphenyl)vinyl]quinolin-8-ol* (**13**). Yield 93% of a beige crystalline compound; Mp. 205-206 °C; E.A. Calcd.: C 31.62%, H 1.59%, N 3.69%; found: C 33.00%, H 2.35%, N 3.72%; HPLC purity 98.58%; UV (nm), λ_max_/log ε: 345.1/3.63; ^1^H-NMR (CDCl_3_), δ: 2.34 (s, 3H, CH_3_), 7.22 (d, *J* = 16.4Hz, 1H, vinyl), 7.85 (d, *J* = 16.4 Hz, 1H, vinyl), 7.40 (s, 1H, quinaldine), 6.61 (d, *J* = 8.7 Hz, 1H, quinaldine), 8.15 (d, *J* = 8.7 Hz, 1H, quinaldine), 7.54 (d, *J* = 8.8 Hz, 1H, phenyl), 7.31 (d, *J* = 2.7 Hz, 1H, phenyl), 6.81 (dd, *J* = 2.7 Hz, *J* = 8.7 Hz, 1H, phenyl); ^13^C-NMR (CDCl_3_), δ: 25.58, 115.27, 117.31, 119.97, 120.64, 121.21, 122.78, 123.24, 124.79, 127.43, 128.00, 129.01, 130.43, 133.97, 138.81, 147.84, 144.99, 152.21, 155.71, 177.45.

*5,7-Dichloro-2-(2-{4-[2-(5,7-dichloro-8-hydroxyquinolin-2-yl)vinyl]phenyl}vinyl)quinolin-8-ol* (**14**). Yield 69% of a dark brown crystalline compound; Mp. 300 °C; HPLC purity 98.68%; UV (nm), λ_max_/log ε: 309.9/3.67 [42].

### 4.3. Lipophilicity determination by HPLC (capacity factor k/calculated log k)

A Waters Alliance 2695 XE HPLC separation module and Waters Photodiode Array Detector 2996 (Waters Corp., Milford, MA, USA) were used. A Symmetry^®^ C_18_ 5 μm, 4.6 × 250 mm, Part No. WAT054275, (Waters Corp., Milford, MA, USA) chromatographic column was used. The HPLC separation process was monitored by Millennium32^®^ Chromatography Manager Software, Waters 2004 (Waters Corp., Milford, MA, USA). A mixture of MeOH p.a. (55.0%) and H_2_O-HPLC– Mili-Q Grade (45.0%) was used as a mobile phase. The total flow of the column was 0.9 mL/min, injection 30 μL, column temperature 30 °C and sample temperature 10 °C. The detection wavelength 210 nm was chosen. The KI methanolic solution was used for the dead time (t_D_) determination. Retention times (t_R_) were measured in minutes.

The capacity factors *k* were calculated using the Millennium32^®^ Chromatography Manager Software according to formula *k* = (t_R_ - t_D_)/t_D_, where t_R_ is the retention time of the solute, whereas t_D_ denotes the dead time obtained via an unretained analyte. Log *k*, calculated from the capacity factor *k*, is used as the lipophilicity index converted to log *P* scale. The log *k* values of the individual compounds are shown in Table 1.

### 4.4. Lipophilicity calculations

Log *P*, *i.e.* the logarithm of the partition coefficient for *n-*octanol/water, was calculated using the programs CS ChemOffice Ultra ver. 10.0 (CambridgeSoft, Cambridge, MA, USA) and ACD/LogP ver. 1.0 (Advanced Chemistry Development Inc., Toronto, Canada). Clog *P* values (the logarithm of *n*-octanol/water partition coefficient based on established chemical interactions) were generated by means of CS ChemOffice Ultra ver. 10.0 (CambridgeSoft, Cambridge, MA, USA) software. The results are shown in Table 1.

### 4.5. In vitro antifungal susceptibility testing

The broth microdilution test [43,44,45] was used for the assessment of *in vitro* antifungal activity of the synthesized compounds against *Candida albicans* ATCC 44859 (CA), *Candida tropicalis* 156 (CT), *Candida krusei* ATCC 6258 (CK), *Candida glabrata* 20/I (CG), *Trichosporon beigelii* 1188 (TB), *Aspergillus fumigatus* 231 (AF), *Absidia corymbifera* 272 (AC), and *Trichophyton mentagrophytes* 445 (TM). Fluconazole (FLU) was used as the standard of a clinically used antimycotic drug. The procedure was performed with twofold dilution of the compounds in RPMI 1640 (Sevapharma a.s., Prague, Czech Republic) buffered to pH 7.0 with 0.165 mol of 3-morpholino-propane-1-sulfonic acid (MOPS, Sigma, Germany). The final concentrations of the compounds ranged from 500 to 0.975 μmol/L. Drug–free controls were included. The MIC determination was performed according to the CLSI (formerly NCCLS) reference protocol M27-A2 for yeasts (IC_80_ value) and M38-A for moulds (IC_50_ value). IC_80_ and IC_50_ were defined as an 80% resp. 50% or greater reduction of growth in comparison with the control. The values of MICs were determined after 24 and 48 h of static incubation at 35 °C. For *T. mentagrophytes*, the final MICs were determined after 72 and 120 h of incubation. The results are summarized in Table 2.

### 4.6. Study of inhibition photosynthetic electron transport (PET) in spinach chloroplasts

Chloroplasts were prepared from spinach (*Spinacia oleracea* L.) according to Masarovicova and Kralova [46]. The inhibition of photosynthetic electron transport (PET) in spinach chloroplasts was determined spectrophotometrically (Genesys 6, Thermo Scientific, USA) using an artificial electron acceptor 2,6-dichlorophenol-indophenol (DCIPP) according to Kralova *et al*. [47] and the rate of photosynthetic electron transport was monitored as a photoreduction of DCPIP. The measurements were carried out in phosphate buffer (0.02 mol/L, pH 7.2) containing sucrose (0.4 mol/L), MgCl_2_ (0.005 mol/L) and NaCl (0.015 mol/L). The chlorophyll content was 30 mg/L in these experiments and the samples were irradiated (~100 W/m^2^) from 10 cm distance with a halogen lamp (250 W) using a 4 cm water filter to prevent warming of the samples (suspension temperature 22 °C). The studied compounds were dissolved in DMSO due to their limited water solubility. The applied DMSO concentration (up to 4%) did not affect the photochemical activity in spinach chloroplasts. The inhibitory efficiency of the studied compounds was expressed by IC_50_ values, *i.e.,* by molar concentration of the compounds causing 50% decrease in the oxygen evolution rate relative to the untreated control. The comparable IC_50_ value for a selective herbicide 3-(3,4-dichlorophenyl)-1,1-dimethylurea, DCMU (Diurone^®^) was about 1.9 μmol/L [48]. The results are summarized in Table 2.

### 4.7. In vitro antimycobacterial evaluation

Clinical isolates of *Mycobacterium avium* complex CIT19/06, *M. kansasii* CIT11/06, *M. absessus* CIT21/06 and strain *M. smegmatis* MC2155 were grown in Middlebrook broth (MB), supplemented with OADC supplement (Oleic, Albumin, Dextrose, Catalase, Becton Dickinson, U.K.). Identification of these isolates was performed using biochemical and molecular protocols. At log phase growth, the 10 mL culture was centrifuged at 15,000 RPM for 20 minutes using a bench top centrifuge (Model CR 4-12 Jouan Inc U.K). Following the removal of the supernatant, the pellet was washed in fresh Middlebrook 7H9GC broth and re-suspended in 10 ml of fresh supplemented MB. The turbidity was adjusted to match McFarland standard No. 1 (3 × 10^8^ cfu) with MB broth. A further 1:20 dilution of the culture was then performed in MB broth.

The antimicrobial susceptibility of all four mycobacteria was investigated in 96 well plate format. Here, 300 µL of sterile deionised water was added to all outer-perimeter wells of the plates to minimize evaporation of the medium in the test wells during incubation. 100 µL of each dilution was incubated with 100 µL of each of the mycobacterial species. Dilutions of each compound were prepared in duplicate. For all synthesized compounds, final concentrations ranged from 300 µg/mL to 10 µg/mL. All compounds were prepared in DMSO and subsequent dilutions were made in supplemented Middlebrook broth. The plates were sealed with parafilm and were incubated at 37 °C overnight in the case of *M. smegmatis *and *M. absessus* and for 5 days in the case of *M. kansasii* and *M. avium* complex. Following incubation, a 10% addition of alamarBlue (AbD Serotec) was mixed into each well and readings at 570 nm and 600 nm were taken, initially for background subtraction and subsequently after 24 hour re-incubation. The background subtraction is necessary with strongly coloured compounds which may interfere with the interpretation of any colour change. In non-interfering compounds, a blue colour in the well was interpreted as an absence of growth, and a pink colour was scored as growth. The MIC was initially defined as the lowest concentration which prevented a visual colour change from blue to pink. The results are shown in Table 3.

The MIC for mycobacteria was defined as a 90% or greater (IC_90_) reduction of growth in comparison with the control. The MIC/IC_90_ value is routinely and widely used in bacterial assays and is a standard detection limit according to the Clinical and Laboratory Standards Institute (CLSI, www.clsi.org/).
